# The cost effectiveness of teriparatide as a first-line treatment for glucocorticoid-induced and postmenopausal osteoporosis patients in Sweden

**DOI:** 10.1186/1471-2474-13-213

**Published:** 2012-10-30

**Authors:** Daniel R Murphy, Lee J Smolen, Timothy M Klein, Robert W Klein

**Affiliations:** 1 Medical Decision Modeling Inc., 8909 Purdue Road, Suite 550, Indianapolis, IN 46268, USA

**Keywords:** Glucocorticoid-induced osteoporosis, Postmenopausal osteoporosis, Cost-effectiveness, Fractures, Teriparatide, Bisphosphonate

## Abstract

**Background:**

This paper presents the model and results to evaluate the use of teriparatide as a first-line treatment of severe postmenopausal osteoporosis (PMO) and Glucocorticoid-induced osteoporosis (GIOP). The study’s objective was to determine if teriparatide is cost effective against oral bisphosphonates for two large and high risk cohorts.

**Methods:**

A computer simulation model was created to model treatment, osteoporosis related fractures, and the remaining life of PMO and GIOP patients. Natural mortality and additional mortality from osteoporosis related fractures were included in the model. Costs for treatment with both teriparatide and oral bisphosphonates were included. Drug efficacy was modeled as a reduction to the relative fracture risk for subsequent osteoporosis related fractures. Patient health utilities associated with age, gender, and osteoporosis related fractures were included in the model. Patient costs and utilities were summarized and incremental cost-effectiveness ratios (ICERs) for teriparatide versus oral bisphosphonates and teriparatide versus no treatment were estimated.

For each of the PMO and GIOP populations, two cohorts differentiated by fracture history were simulated. The first contained patients with both a historical vertebral fracture and an incident vertebral fracture. The second contained patients with only an incident vertebral fracture. The PMO cohorts simulated had an initial Bone Mineral Density (BMD) T-Score of −3.0. The GIOP cohorts simulated had an initial BMD T-Score of −2.5.

**Results:**

The ICERs for teriparatide versus bisphosphonate use for the one and two fracture PMO cohorts were €36,995 per QALY and €19,371 per QALY. The ICERs for teriparatide versus bisphosphonate use for the one and two fracture GIOP cohorts were €20,826 per QALY and €15,155 per QALY, respectively.

**Conclusions:**

The selection of teriparatide versus oral bisphosphonates as a first-line treatment for the high risk PMO and GIOP cohorts evaluated is justified at a cost per QALY threshold of €50,000.

## Background

Osteoporosis is a common disease that is characterized by a significant loss of bone mass. While some gradual loss of bone mass occurs in most people as they age, those with osteoporosis see an accelerated loss. The impact of this accelerated loss is a substantially higher risk of bone fractures. Because osteoporosis has no symptoms, a diagnosis frequently occurs only after a fracture event.

Osteoporosis related fractures have a significant impact on quality of life, medical costs, and mortality. A 2007 study by Borgström et al. [1] estimated the costs of osteoporosis in Sweden at SEK 8.5 billion annually. Statistics from Kanis et al. [2] indicate that the chance of an osteoporosis related hip fracture in a 50 year old Swedish woman (in their remaining lifetime) was 22.9%. Another analysis from Kanis et al. [3] indicates that mortality risk after an osteoporosis related hip fracture increases by 20 to 30 percent for three to six months after the event solely due to the fracture.

Treatment for osteoporosis often follows diagnosis after a fracture event, but may be initiated earlier if risk factors for a diseased patient are identified. A bone mineral density (BMD) test is the standard diagnostic tool to confirm the condition. The test measures the density of minerals (such as calcium) using dual energy X-ray absorptiometry (DXA) or computed tomography (CT) scan. The test output is called a T-score. A T-score is defined as the difference, reported in units of standard deviation (SD), between the individual’s measured BMD and that of a healthy young adult. A test result indicating more than −2.5 SD from the reference mean value is defined as clinical osteoporosis.

Among osteoporosis patients two disease subgroups were identified for analysis within this study:

1. Postmenopausal osteoporosis (PMO) patients [4-7] – Postmenopausal women represent the largest group of osteoporosis patients.

2. lucocorticoid-induced osteoporosis patients (GIOP) [8] – GIOP is the most common cause of secondary osteoporosis [9]. Glucocorticoids (e.g., prednisone) are typically used to treat rheumatoid arthritis, lupus, myositis, and polymyalgia rheumatica. GIOP patients are at very high risk of fractures through the depletion of bone mass [10,11].

First-line pharmaceutical treatment for both PMO and GIOP patients typically involves one of several oral bisphosphonates [12]. In normal adults, bone mass is continually being created and reabsorbed by the body. Bisphosphonates work by slowing the rate at which bone is reabsorbed. For severe PMO and GIOP patients, however, slowing bone reabsorption may not be sufficient in reducing the risks of osteoporosis related fractures. Unlike bisphosphonates, teriparatide acts by stimulating osteoblasts, increasing the number and function of osteoblasts and leading to new bone formation. When bisphosphonates are insufficient, the use of teriparatide may be indicated.

Past studies have indicated that teriparatide is more clinically efficacious than bisphosphonates for PMO and GIOP patients [8]. Bisphosphonates, however, represent a significantly lower (direct) cost treatment option. This poses the primary objective of this cost effectiveness analysis; to compare teriparatide in first-line use against bisphosphonates and against no treatment in two high risk cohorts. Our goal in doing so was to evaluate under what conditions teriparatide may represent a cost effective first-line option.

## Methods

### Cost effectiveness model

The study used a java based microsimulation model to estimate patient events, costs, utilities, and cost-effectiveness. The model simulates the lives of patients from a given starting age until death or until the end of follow-up. Hip fractures, clinical vertebral fractures, and wrist fractures were modeled. The modeled mortalities were population-based natural mortality and excess mortality due to fracture. The model has a cycle length of six months. During each cycle, the simulated patients were at risk of both osteoporosis related fractures and death.

**Figure 1:**
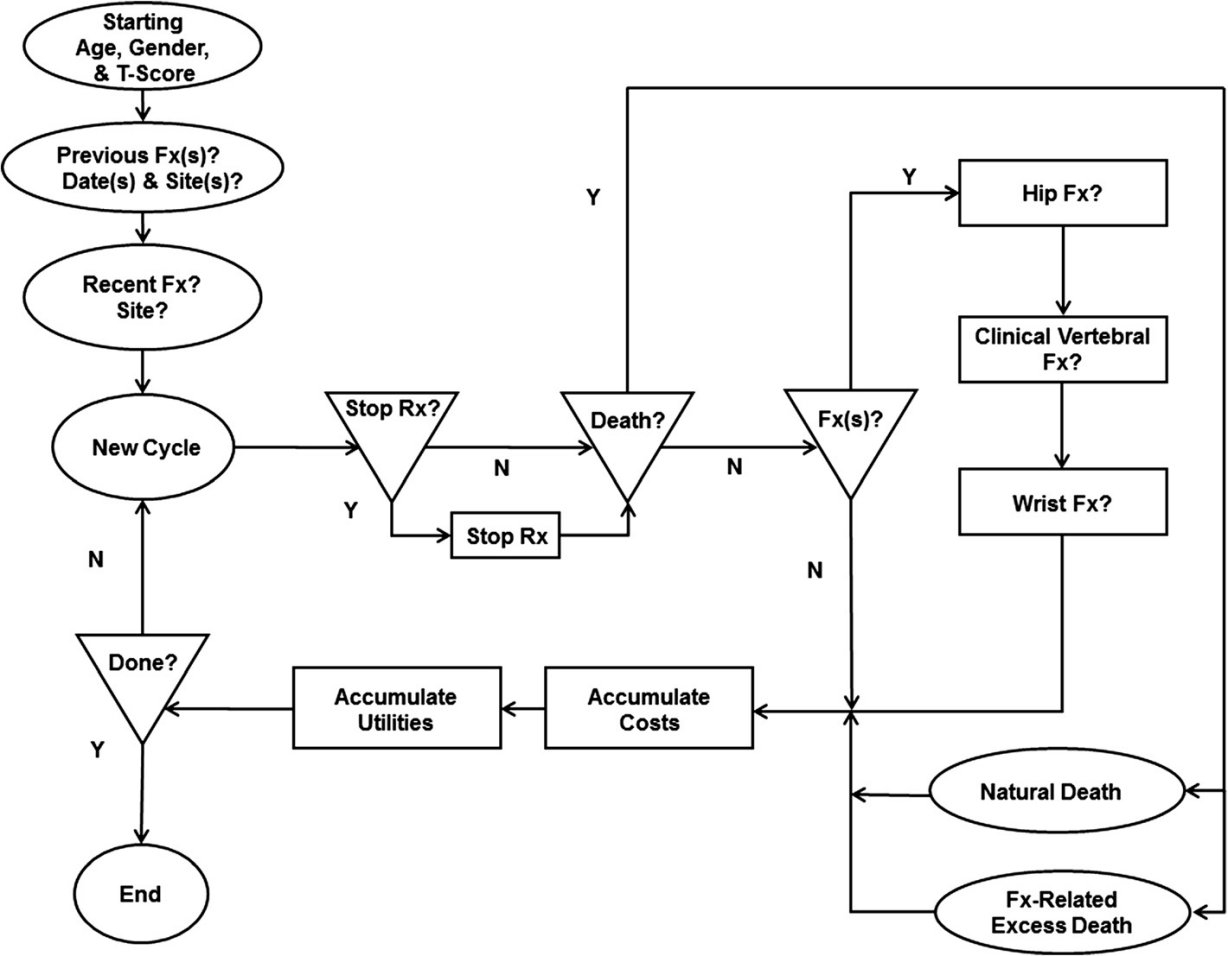
Model Flow for Patient Treatment (Rx) and Fracture Generation (Fx).

The model is implemented as a Monte Carlo simulation: a large number of individual patients are stochastically processed through the model. Identical cohorts of patients are simulated by the model to compare costs and benefits for teriparatide treatment, bisphosphonate treatment, and no treatment. The simulated patients are defined by a set of initial characteristics. The primary characteristics are the patient’s starting age, gender, and initial BMD T-score. The base case analyses were performed using 200 replications of 100,000 simulated patients. Results are saved for each patient and the mean expected cost and effectiveness for each cohort is calculated. The basic flow of patients within the model is shown in Figure 1.

Costs estimated by the model included those associated with treatment and with fracture events. Patient utility values were also estimated by the model. These were age-dependent values that were multiplied accordingly by fracture site-specific utility multipliers, both in the six month cycles in which the fractures occurred and in subsequent cycles. Costs and utilities were reduced in the six month cycles in which death occurred. Both costs and benefits were discounted at annual discount rates of 3%. Model results were analysed by comparing the average cost and utility-determined quality-adjusted life year (QALY) values for the three treatment options. The model also estimated the cohort’s undiscounted life years associated with each treatment option.

### Patient cohorts

For the analysis of teriparatide treatment in PMO patients, two patient cohorts were simulated:

• Patients with a BMD T-score of −3.0 SD below the mean, a historical vertebral fracture (5 years previous) and an incident vertebral fracture (6 months previous) using teriparatide as a first-line treatment; and

• Patients with a BMD T-score of −3.0 SD below the mean and an incident vertebral fracture using teriparatide as a first-line treatment.

In both PMO cohorts females were evaluated with treatment starting at age 69. The patient age, fracture history, and BMD used for this analysis were selected to represent the prime years of recognition and treatment for a severe PMO case requiring immediate and aggressive treatment. The inclusion of both a historical and an incident vertebral fracture in the first patient cohort is reflective of reimbursement criteria for PMO patients in Sweden. Criteria vary between countries, with many requiring three or more previous fractures based on patient age and T-score.

For the analysis of teriparatide treatment in GIOP patients, two cohorts were examined:

• Patients with GIOP, a BMD T-score of −2.5 SD below the mean, a historical vertebral fracture (5 years previous) and an incident vertebral fracture using teriparatide as a first-line treatment, and

• Patients with GIOP, a BMD T-score of −2.5 SD below the mean, and an incident vertebral fracture using teriparatide as a first-line treatment.

The GIOP cohorts evaluated also started as 69 year old patients. They are a combination of males and females that reflect the gender proportion of patients with GIOP. The assumption of 80% female GIOP patients was based on the participant characteristics of a published clinical trial assessing the benefit of treatment with teriparatide for patients with GIOP [8].

### Model data sources

A summary of data sources used in the model is presented in Table 1. A detailed listing of model data and references is provided in Additional file 1: Appendix A, Table A1.

**Table 1:** Key model parameters

**Parameter**	**Value**	**References**
Baseline Fracture Risks	based on Fx type, age, and gender, BMD	[2,13]
Fracture Relative Risks - GIOP	by fracture type based on age	[13]
Fracture after Fracture and Second Fracture Relative Risks	based on fracture type and age	[13,14]
Drug Costs	Teriparatide- Avg. Daily Cost: €14.74	[15]
	Alendronate – Avg. Daily Cost: €0.42	
Acute and Long-Term Direct Fracture Costs	by age and fracture type	[14,28]
Currency SEK to € Conversion	9.0335 ; 12 month average ending December 2011	[16]
Continuing 6 Month Care Costs	€201 (months 1–6,13-18)	[17]
	€194 (months 7–12)	
Natural Mortality	Swedish Life Tables based on age and gender; adjusted to remove osteoporosis fracture deaths	[13,18]
Fracture Mortality	based on age and gender;	[13]
	0.007 (65 year male) to 0.0375 (90+ year old female)	
Relative Risk of Mortality Post Fracture	For hip fractures and vertebral fractures; ranges: 2.5 (year 1) to 1.0 (year 7)	[19,20]
Base Health Utilities	by age; from 0.84 (through age 50) to 0.65 (age 85)	[21]
Fracture Utility Multipliers	first year and subsequent years, by fracture type	[13]
Teriparatide Anti-Fracture Efficacies Relative Risks	by fracture type and treatment period	[4-7]
Bisphosphonate Anti-Fracture Efficacies Relative Risks	by fracture type and treatment period	[13]
Teriparatide Discontinuation	3, 9, and 15 month values interpolated to 6 month cycles	[22]
Bisphosphonate Discontinuation	by 6 month cycle	[23]

### Fracture risks

Fracture risks in the model were based on age; gender; mean and measured T-scores; the timing and type of previous fractures; glucocorticoid use; and treatment. The algorithm implemented for the calculation of fracture risk is from a systematic review [13] addressing the treatment of GIOP. Relative fracture risks were applied to the baseline risks to compensate for the additional risks associated with previous fractures and glucocorticoid use. Treatment-dependent relative risks (or therapy fracture efficacy rates) were applied to obtain the fracture risks with treatment. The treatment-dependent relative risks were applied during the course of treatment and were phased out after the completion (or cessation) of treatment.

First fracture risks for Swedish females with average BMD values were adjusted for age. The ratios of UK male to female fracture risks from Kanis (Table twenty) [13] were applied to the Swedish female fracture risks to impute the corresponding fracture risks for Swedish males. The model assigns the fracture risks to the midpoint of the stated age ranges, e.g., the 50–54 fracture risk values were assigned to 52 year olds. Fracture risks for other ages were interpolated from the table values.

Baseline fracture risks for a given GIOP patient were calculated by applying an additional relative risk factor [13]. The additional relative risk factor reflects the increased risk of fracture due to prior or current glucocorticoid use.

Fracture risks were also dependent on previous fractures [14]. The base model assumed that the relative risk of fracture after a previous fracture was not age-dependent. The base model also assumed that the impact on fracture risk from a previous fracture continued for a period of 5 years after each fracture. The model has the capability to modify this post-fracture impact timeframe, ranging from no post-fracture impact to patient lifetime post-fracture impact.

In the case of multiple historical fractures of different types, the model utilizes the largest relative risk of fracture multiplier. The model includes an additional risk due to a second fracture of each type. This was set as an additional percentage of the risk increase due to the first fracture (50% increase for GIOP patients and 100% for PMO patients).

### Fracture costs

Costs of a fracture were divided into acute costs, which occur during the first six-month cycle following the fracture, and continuing long-term costs which are modeled as persisting for the remainder of the patient lifetime. Where data were available, costs were differentiated by age groups. Fracture costs were summed for a model cycle. Cycle costs for a given model cycle may include multiple acute six-month costs for different fracture types, as well as multiple continuing costs from previous fractures.

Acute fracture costs are accumulated during the first six months after hip, clinical vertebral, and wrist fractures. Continuing costs accrue in post-fracture cycles for the lifetime of the patient. Wrist fractures are assumed to result in no continuing costs. For hip fractures, continuing costs are based on the cost of nursing home care and the likelihood that the fracture resulted in admittance to a nursing home. Continuing costs for clinical vertebral fractures include costs for each six months of analgesic usage.

For a repeat occurrence of a fracture event with long-term continuing costs, the model adds the acute six-month costs of the fracture event, and then subsequently uses only the continuing costs of the repeat fracture (thus avoiding potential double-counting of long-term care costs). This approach also allows for the higher long-term continuing costs associated with older patients to supersede lower long-term continuing costs from previous fractures. In the rare cases where the previous fracture event’s long-term continuing costs are higher, the higher costs are accumulated.

### Drug intervention, physician, and testing costs

Patients were assumed to be treated either with teriparatide for 18 months, with bisphosphonates for 18 months, or with no treatment. Drug costs came from the Swedish Dental and Pharmaceutical Benefits Agency (TLV) historical database on pricing decisions [15]. The daily cost of teriparatide was set as €14.74, representing the public price in Sweden of a teriparatide pen (28 injections for 3,728.50 SEK). The daily bisphosphonate intervention cost was set as €0.42, based on the price for generic 10 mg daily alendronate (98 tablets for 372.00 SEK). Note that a weekly dose of alendronate at a slightly higher daily cost (based on the TLV database) is also available. The lower cost option, least favourable to teriparatide, was selected for this analysis.

Costs for physician visits and BMD testing were taken from the Swedish 2011 Regional Rates and Allowances for Southern Health Region price list [17]. For visits involving a BMD test, the cost was set as the price for an osteoporotic measurement visit (in Malmo) of 1,125 SEK plus the cost of a hand BMD measurement of 695 SEK. For visits not involving a BMD measurement, the cost was set as the basic rate in Malmo for the most recent doctor seen by the patient, 1,756 SEK. Patient visits (and their costs) were assigned for every six-month cycle that a simulated patient receives treatment. Over 18 months of teriparatide or bisphosphonate therapy, this results in 3 visits for adherent patients. The cost for BMD measurements was assigned to treated patients in the first six-month cycle and subsequently every other six-month cycle. The base case analysis has no side effect costs for treatment with teriparatide or bisphosphonates.

### Treatment discontinuation

The model estimates cycle-by-cycle treatment discontinuation. For the base case analysis, teriparatide discontinuation of treatment was based on European Forsteo Observational Study (EFOS) data from Langdahl [22] (Figure 1). The discontinuation of bisphosphonate treatment was based on time to persistence failure data reported by Weycker [23].

### Mortality

The model simulates both natural (i.e., not fracture-related) mortalities and fracture-related excess mortalities. The age-gender-specific mortality rates for the general population in Sweden were based on data from the Swedish Life Tables for years 2003–2007 [18]. In order to avoid the double-counting of fracture-related excess mortalities, the proportions of deaths attributable to fracture from Kanis [13] (Tables twenty seven and twenty nine) were removed from the Swedish mortality rates to obtain the natural mortality rates without fractures. For the accumulation of treatment intervention costs, fracture event costs, and health state utilities, natural mortalities were assumed to occur at the mid-point of the six-month cycle, while fracture-related excess mortalities were assumed to occur at the start of the cycle.

Mortality risk has been found to increase after hip and vertebral fractures [19,20,24]. Wrist and other types of fractures were assumed not to affect mortality. These multipliers are dependent on time since hip or vertebral fractures: they were phased out over 6 years for vertebral fractures and 7 years for hip fractures. If there were both vertebral and hip fractures within the last 6 years, the model used the larger risk multiplier.

### Utilities

Health utility is known to be partly dependent on age and gender. Base utilities used in the model were taken from Swedish population norms found in Lundberg [21]. The reductions in health utilities after osteoporotic fractures were based on Kanis [13], (Table thirty). A multiplier was used to calculate the utility weight after a fracture, which implied that all fractures produce a percentage decline in health utility compared with the age-specific average for healthy patients.

Health utility calculations in the model for all fractures were based on the assumption that reductions in health utility from fractures are multiplicative. If more than one fracture occurred during the same six month cycle, the health utility was estimated to be the product of the multipliers for each fracture type and the base utility. Similarly, the reduction in health utility for previous prevalent fracture and the acute reduction in health utility for a new fracture were multiplicative. For example, a clinical vertebral fracture in a 60 year-old male with a previous hip fracture would have a utility of 0.412 (calculated as 0.81_[base]_ * 0.626_[first-year vertebral]_ * 0.813_[subsequent hip]_) or about a 49% reduction in health utility during the first year after fracture.

For repeat occurrences of a particular type of fracture, the model used the first-year utility multiplier in the year of the repeat fracture and a specified proportion (25% by default) of the disutility attributable to the subsequent utility multiplier from the previous fracture. This produced results which were consistent with lower utilities for multiple fractures of the same type identified in the Lips [25] review of studies of quality of life in osteoporosis.

### Teriparatide efficacy

The efficacy of the intervention was measured as relative risk reduction to fracture rates compared to no treatment. For this model, these relative risk reductions were based on clinical trials dealing with therapeutic interventions reported in Neer [4], Lindsay [5], Prince [6], Genant [7], and Kanis [13].

Neer [4] and Genant [7] described the use of teriparatide versus placebo in the treatment of osteoporosis in postmenopausal women. These efficacies were also used as representative of the teriparatide efficacies in the treatment of GIOP. The model incorporated the clinical vertebral fracture relative risk results for teriparatide versus placebo (RR = 0.17) from Genant [7]. The model incorporated the non-vertebral fracture (pooled hip, wrist, ankle, humerous, rib, foot, pelvis and other) relative risk results for teriparatide versus placebo (RR = 0.47) from Neer [4]. These relative risks were held constant for the 18 months of treatment.

The model used a 57% relative risk reduction (RR = 0.43) for clinical vertebral fractures compared to no treatment for the 18-month period after discontinuation of treatment with teriparatide. The risk reduction was based on the sustained efficacy for the prevention of moderate or severe vertebral fractures demonstrated by teriparatide during an 18-month period after discontinuation of treatment from Lindsay [5]. Mild vertebral fractures are usually asymptomatic and therefore rarely receive clinical attention. As the model simulated only clinical vertebral fractures, the risk reduction of moderate and severe vertebral fractures in postmenopausal women was used as a proxy for clinical vertebral fractures.

The model applied a 27% relative risk reduction (RR = 0.73) for hip and wrist fractures during the 30-month period after discontinuation of teriparatide treatment from Prince [6]. The results reported in Prince from the Fracture Prevention Trial (FPT) were not powered to isolate specific non vertebral fractures (e.g., hip fractures), so a pooled RR reduction was applied to the group. The fracture prevention efficacy of teriparatide was modeled to decline linearly over an 18-month period after the end of the sustained efficacy period.

### Bisphosphonate efficacy

The model used a set of combined bisphosphonate anti-fracture efficacies from the Kanis [13] study of GIOP treatment. In that study, Kanis based those therapy efficacies on two assumptions; (1) responses to intervention did not differ between the bisphosphonates, and (2) fracture risk reductions were similar for patients taking glucocorticoids as for women with postmenopausal osteoporosis. The current model implicitly used these same assumptions.

The relative risks associated with bisphosphonate treatment were held constant for the 18 month treatment cycle. After completion of treatment, the relative risks were ramped back to no benefit over an 18-month period. This was based on the supposition that the treatment efficacy of bisphosphonates is expected to decline to zero during a period equal to the duration of treatment after the discontinuation of treatment from Schousboe [26].

### Currency conversions and base year

Conversions from Swedish currency to euros were performed using the exchange rate of 9.0335 SEK per euro, which was the average exchange rate over the previous 12 months ending December 2011 [16]. All non 2011 costs were inflated from their base year to January 2012 based on Sweden historical consumer price index (CPI) values from Statistics Sweden [27].

### Sensitivity analyses

One-way sensitivity analysis and probabilistic sensitivity analysis (PSA) were performed for all cohorts. Only sensitivity analyses for the 2-fracture cohorts were reported in this manuscript.

For the one-way sensitivity analysis, individual model input variables representing assumptions about treatment efficacies, population fracture-related rates, epidemiologic and demographic data, and economic variables of interest were varied over specified ranges that reflected uncertainties in their point estimates. Results are displayed as tornado diagrams.

For the probabilistic sensitivity analysis, 1,000 simulations of 1,000 patients were performed on each cohort. For each simulation, the values of the critical parameters were sampled from distributions. For this analysis, those parameters were fracture treatment costs, fracture related utilities, and drug efficacies in reducing fractures. Scatter plots of incremental QALYs by incremental costs and acceptability curves showing the probability that the cost per QALY is less than various willingness-to-pay thresholds are displayed for both 2-fracture cohorts.

For all of the specified distributions, the mean values were set equal to the baseline estimate values. Fracture costs were represented by lognormal distributions, while the disutility due to fractures and fracture risk reductions due to treatment were represented by beta distributions. Fracture costs were allowed to vary from 50% to 200% of baseline. Fracture (dis)utilities were allowed to range between the minimum and maximum disutility values found in Schousboe [28] for each fracture type. Fracture risk reductions due to treatment were allowed to vary within the 95% confidence interval from the teriparatide clinical trials [4,7]. A detailed listing of the probabilistic sensitivity analyses parameters is provided in Additional file 1: Appendix A, Table A2.

The threshold for cost-effectiveness was set at €50,000 (451,675 SEK) throughout these results.

## Results and discussion

### Base case results

The major results for both the PMO and GIOP cohorts evaluated are presented in Tables 2, 3, and 4. Clinical fracture results estimated from the model are displayed in Table 2. The results show fractures avoided per 1,000 patients for teriparatide against no treatment and teriparatide against bisphosphonate. Each cohort evaluates two cases: (1) patients with an historical and an incident fracture and (2) patients with an incident fracture only.

**Table 2:** Base case clinical results: fractures avoided per 1,000 patients

**Cohort Evaluated**	**Hip Fractures**	**Vertebral Fractures**	**Wrist Fractures**
**PMO Patients (100% Female), age 69 years, T-Score = −3.0**			
historical vertebral + incident vertebral fracture			
Teriparatide vs. No Treatment	95	221	32
Teriparatide vs. bisphosphonate	66	166	27
incident vertebral fracture			
Teriparatide vs. No Treatment	73	145	27
Teriparatide vs. bisphosphonate	51	108	22
**GIOP Patients (80% Female), age 69 years, T-Score = −2.5**			
historical vertebral + incident vertebral fracture			
Teriparatide vs. No Treatment	93	304	36
Teriparatide vs. bisphosphonate	63	227	30
incident vertebral fracture			
Teriparatide vs. No Treatment	85	248	34
Teriparatide vs. bisphosphonate	57	184	28

**Table 3:** Base case clinical results: incremental life years per 1,000 patients

**Cohort Evaluated**	**Incremental Life Years**
**PMO Patients (100% Female), age 69 years, T-Score = −3.0**	
historical vertebral + incident vertebral fracture	
Teriparatide vs. No Treatment	132
Teriparatide vs. bisphosphonate	102
incident vertebral fracture	
Teriparatide vs. No Treatment	102
Teriparatide vs. bisphosphonate	77
**GIOP Patients (80% Female), age 69 years, T-Score = −2.5**	
historical vertebral + incident vertebral fracture	
Teriparatide vs. No Treatment	153
Teriparatide vs. bisphosphonate	117
incident vertebral fracture	
Teriparatide vs. No Treatment	140
Teriparatide vs. bisphosphonate	106

**Table 4:** Base case cost effectiveness results (with 95% CIs)

**Cohort Evaluated**	**Teriparatide vs. No Treatment**	**Teriparatide vs. Bisphosphonate**
**PMO Patients (100% Female), age 69 years, T-Score = −3.0**		
historical vertebral + incident vertebral fracture	€5,897 / QALY	€19,371 / QALY
	(€5,128 – €6,612)	(€18,413 – €20,424)
incident vertebral fracture	€18,701 / QALY	€36,995 1 / QALY
	(€17,612 – €20,062)	(€35,252 – €38,944)
**GIOP Patients (80% Female), age 69 years, T-Score = −2.5**		
historical vertebral + incident vertebral fracture	€3,271 / QALY	€15,155 / QALY
	(€2,691 – €3,853)	(€14,406 – €15,881)
incident vertebral fracture	€ 7,330/ QALY	€20,826 / QALY
	(€6,650 – €8,062)	(€19,831 – €21,854)

Looking at Table 2, the results indicate a reduction in fractures rate per 1,000 patients across both cohorts with teriparatide use, which is consistent with its expected efficacy over bisphosphonate. Depending on the cohort and fracture history, these rates indicate that teriparatide treatment would prevent between 245 to 433 fractures over no treatment and between 181 and 320 fractures over bisphosphonate treatment per 1,000 patients.

Table 3 presents the incremental undiscounted life years associated with both cohorts. The data indicates that the reduction of osteoporosis related fractures improves the lifespan of both cohorts (by reducing fracture related mortality). This benefit ranges from 102 to 153 additional life years per 1,000 patients for teriparatide against no treatment and 77 to 117 additional life years per 1,000 patients for teriparatide against bisphosphonate treatment.

Table 4 presents the base case incremental cost effectiveness ratios for the cohorts evaluated. The model estimates ICERs less than €50,000 per discounted QALY in all cases. The ICERs estimated for the GIOP cohorts are all smaller than those estimated for the PMO cohorts with identical fracture profiles, even though a T-score of −2.5 was evaluated against a T-score of −3.0 for the PMO cohorts. This indicates that the additional fracture risks associated with the GIOP cohort were greater than the reduced fracture risks due to the more favourable BMD score.

Detailed simulation results showing estimated fractures, costs, QALYs, and life years for both cohorts are presented in Additional file 2: Appendix B.

### PMO Patients: one-way sensitivity analyses and PSA results

**Figure 2:**
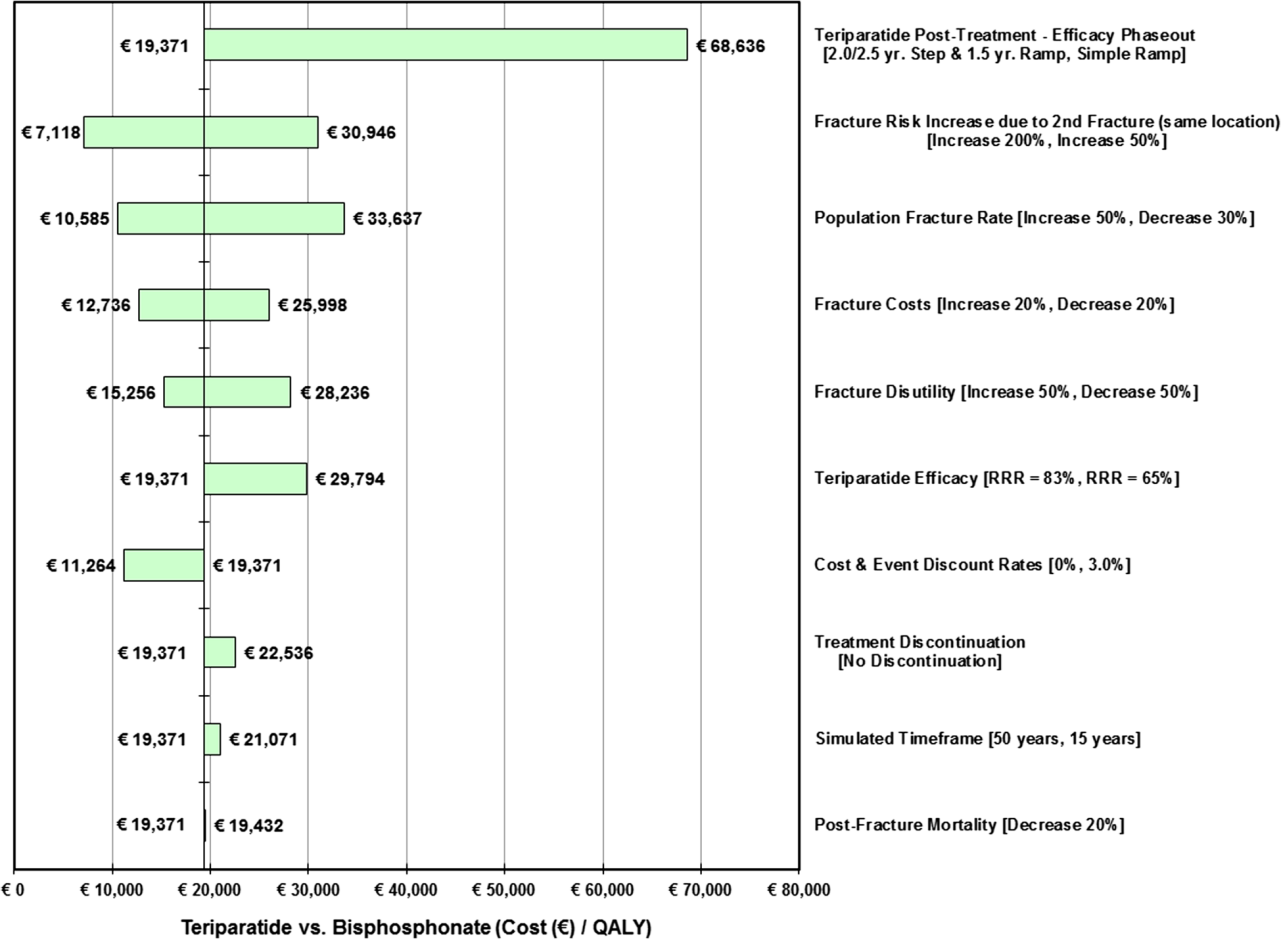
One-Way Sensitivity Analysis Tornado Diagram: Teriparatide vs. Bisphosphonate ICERs for the PMO T −3.0/2 Fracture Cohort.

Results from the one-way sensitivity analysis for the PMO T-3.0/2 fracture cohort are displayed in Figure 2. The specific values changed for the analyses are shown in the figure. These results indicate that the variations made to every (single) major model parameter examined, except a linear teriparatide phase out period equal to the duration of therapy, will generate Cost / QALY estimates for teriparatide vs. bisphosphonate which are less than the €50,000 per QALY threshold.

**Figure 3:**
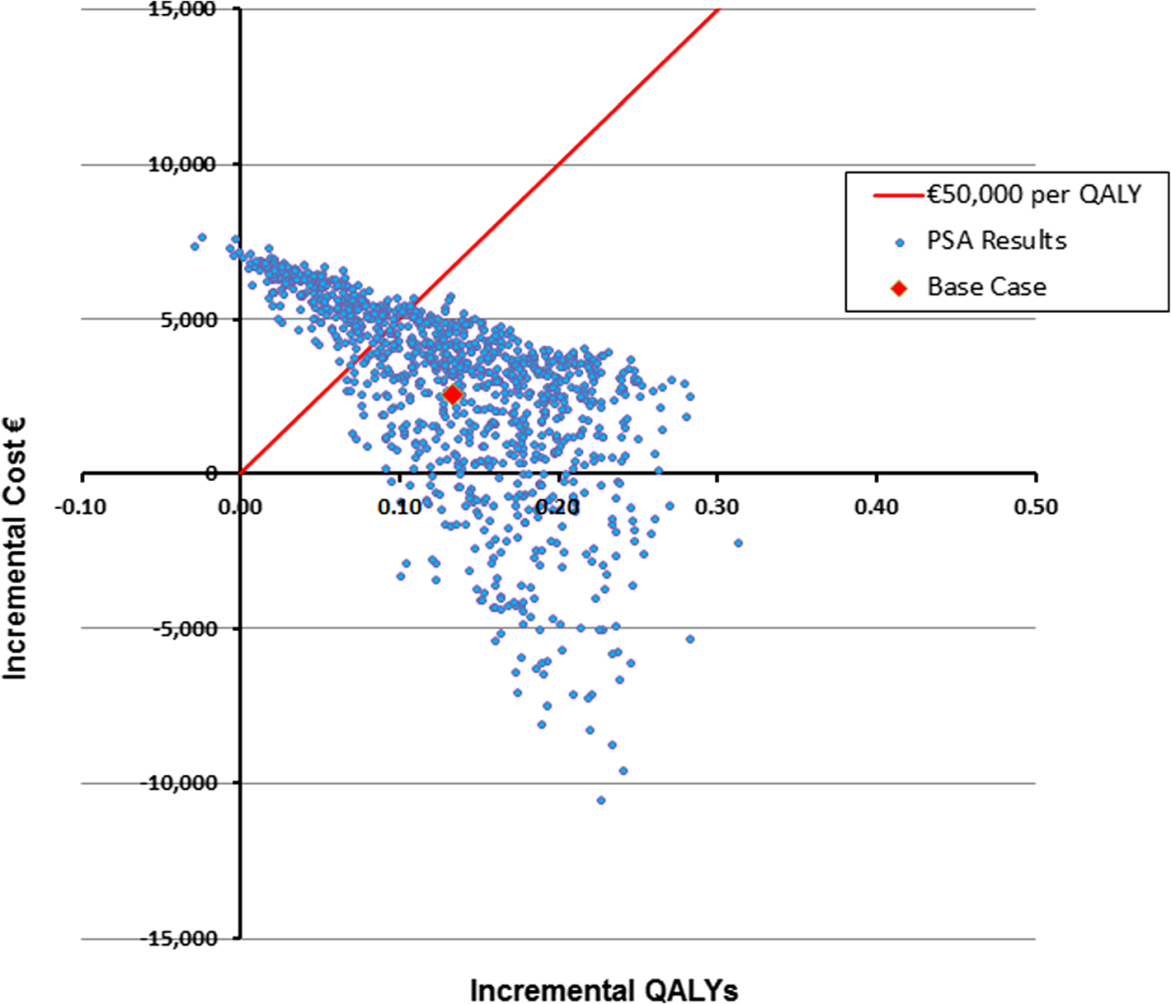
Scatter Plot of Incremental Costs and QALYs for PMO T −3.0/2 Fracture Cohort: Teriparatide vs. Bisphosphonate Treatment.

Figure 3 presents PSA results for teriparatide versus treatment with bisphosphonate for the PMO cohort with a historical and an incident vertebral fracture. The scatter plot shows the mean incremental costs and QALYs for 1,000 simulations, each with 1,000 patients. The mean incremental cost and QALY for the base case results for this cohort from Table 4 is also displayed, as well as the €50,000 per QALY threshold.

From the 1,000 PSA simulations for the PMO −3.0, 2-fracture cohort, 829 produced results with positive incremental costs and positive incremental QALYs. 165 simulations estimated positive incremental QALYs for teriparatide use versus bisphosphonate use with less incremental costs (i.e., producing dominant results for teriparatide); and 6 simulations estimated negative incremental QALYs for teriparatide use with greater incremental costs than bisphosphonate use (i.e., producing dominant results for bisphosphonate use).

**Figure 4:**
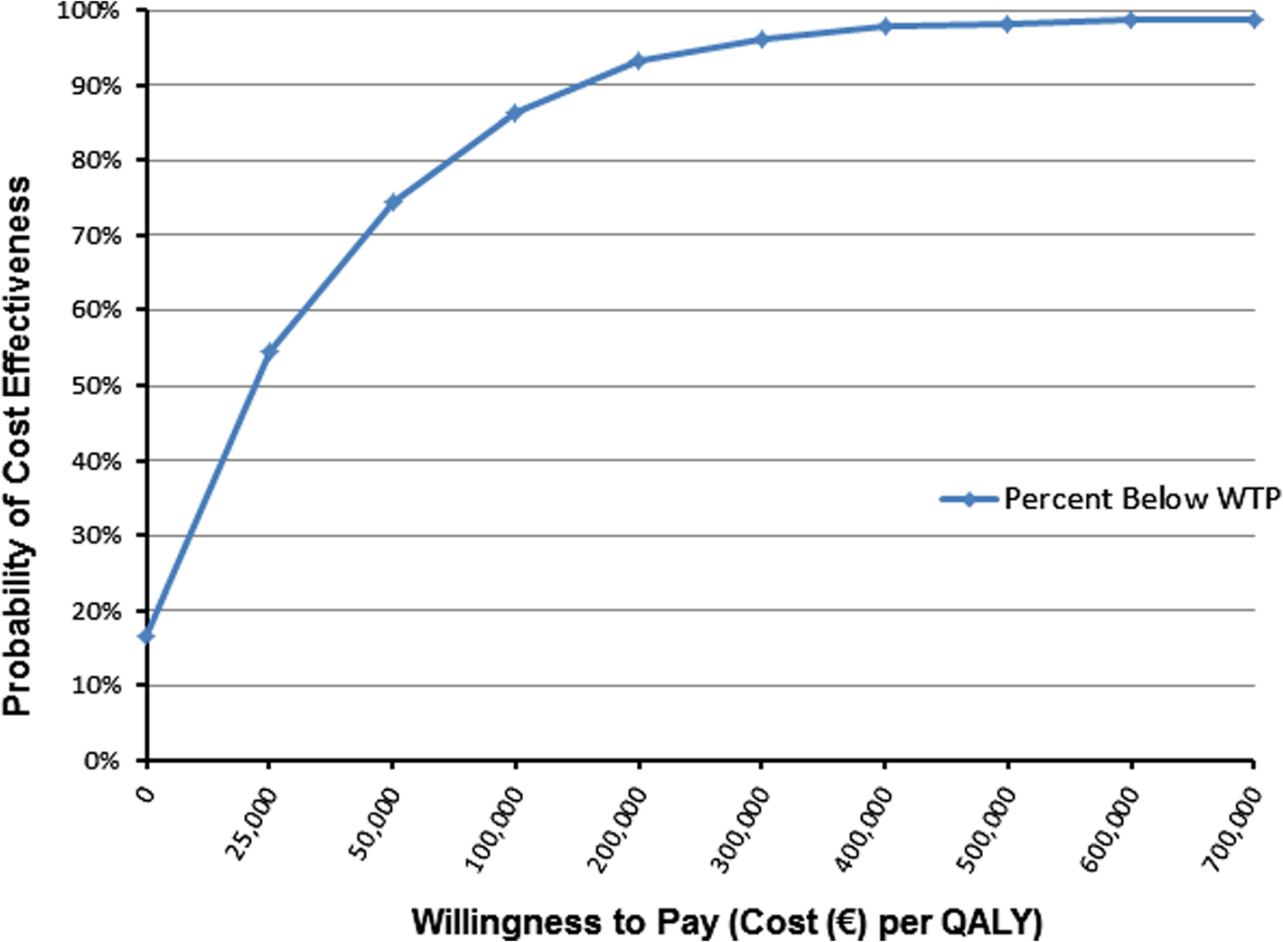
Acceptability Curve for PMO T −3.0/2 Fracture Cohort: Teriparatide vs. Bisphosphonate Treatment.

The acceptability curve for the PMO T-3.0/2 fracture cohort is shown in Figure 4 for teriparatide against bisphosphonate treatment. This curve was also based on total incremental costs and QALYs from 1,000 simulations of 1,000 patients. From the curve, it can be seen that 74.3% of the simulations produced mean incremental costs per QALY less than €50,000.

### GIOP patients: one-way sensitivity analyses and PSA results

**Figure 5:**
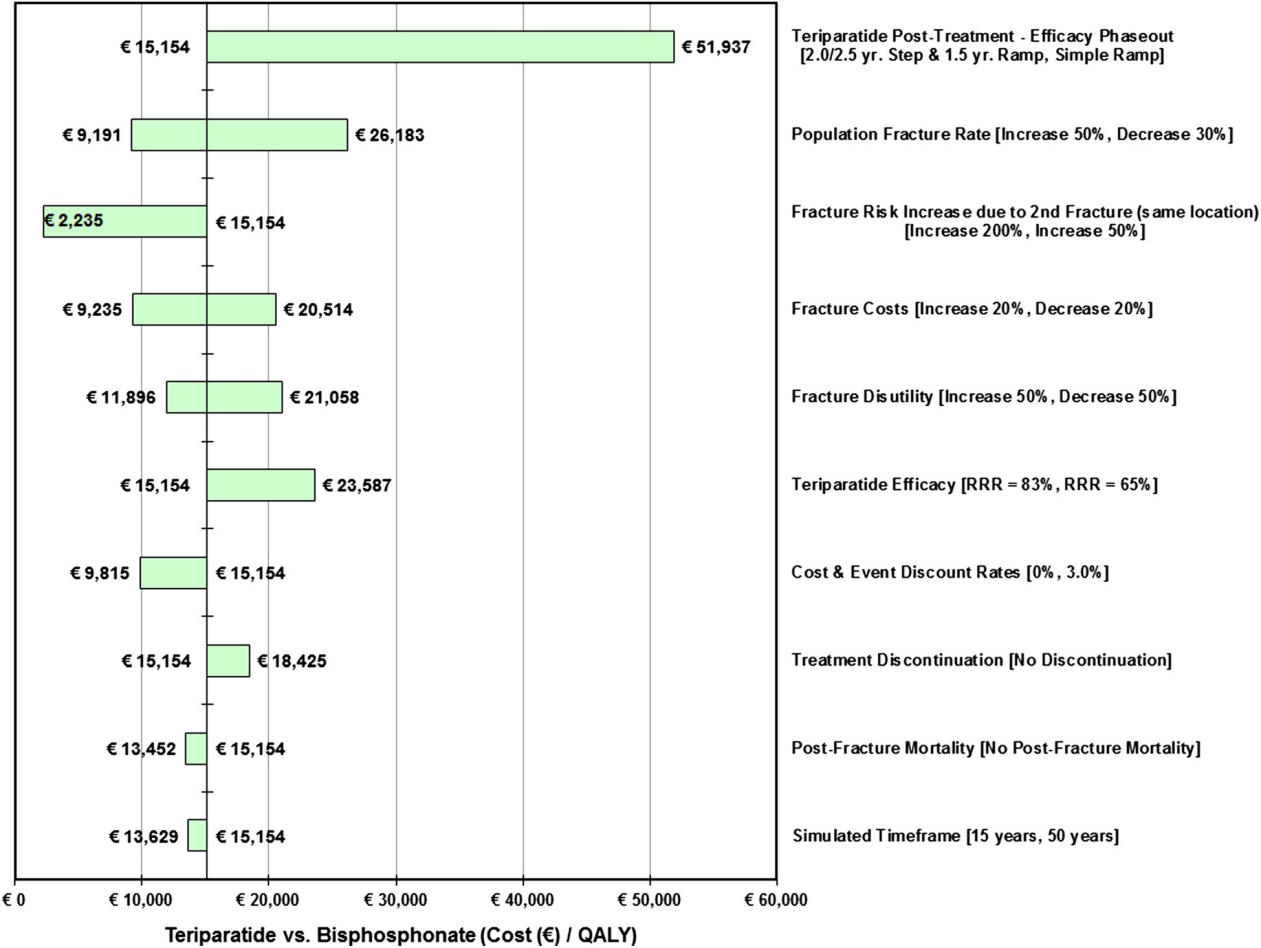
One-Way Sensitivity Analysis Tornado Diagram: Teriparatide vs. Bisphosphonate ICERs for the GIOP T −2.5/2 Fracture Cohort.

Results from the one-way sensitivity analysis for the GIOP T-2.5/2 fracture cohort are displayed in Figure 5. The specific values changed for the analyses are shown in the figure. As with the PMO SA, the GIOP results indicate that variations to (single) major model parameters, except a linear phase out of teriparatide efficacy equal to the duration of therapy, will generate an ICER for teriparatide over bisphosphonate use at less of €50,000 per QALY.

**Figure 6:**
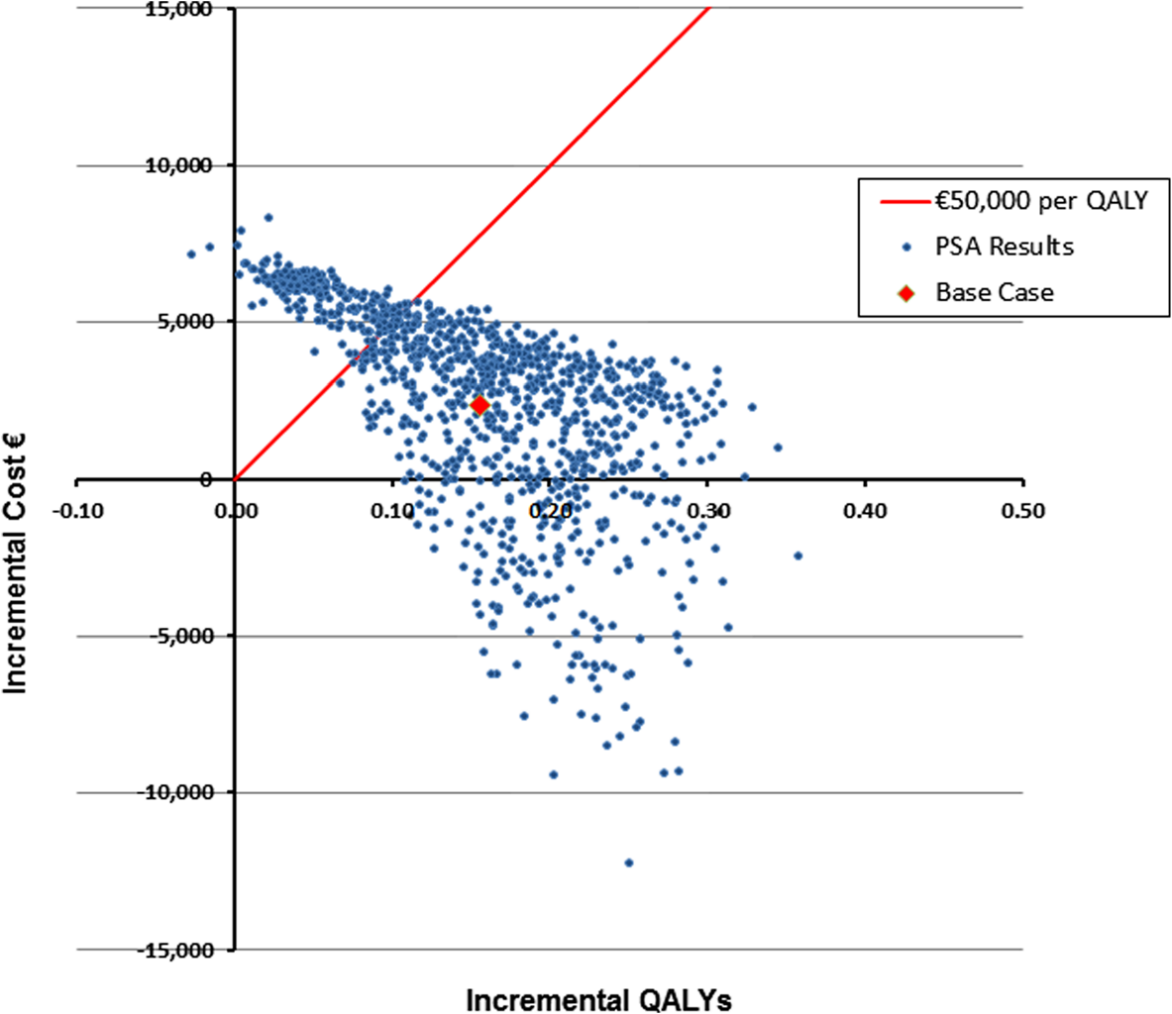
Scatter Plot of Incremental Costs and QALYs for GIOP T −2.5/2 Fracture Cohort: Teriparatide vs. Bisphosphonate Treatment.

Figure 6 presents PSA results for teriparatide versus treatment with bisphosphonate in the GIOP cohort with T-scores of −2.5 and a historical vertebral fracture and incident vertebral fracture. From Figure 6, the incremental cost per QALY for teriparatide versus bisphosphonate treatment was less than the €50,000 threshold in 79.9% of 1,000 simulations for these GIOP patients.

Out of the 1,000 PSA simulations for the GIOP T −2.5/2 fracture cohort, 810 produced results with positive incremental costs and positive incremental QALYs. 188 simulations estimated positive incremental QALYs for teriparatide use versus bisphosphonate use with less incremental costs (i.e., producing dominant results for teriparatide); and 2 simulations estimated negative incremental QALYs for teriparatide use with greater incremental costs than bisphosphonate use (i.e., producing dominant results for bisphosphonate use).

**Figure 7:**
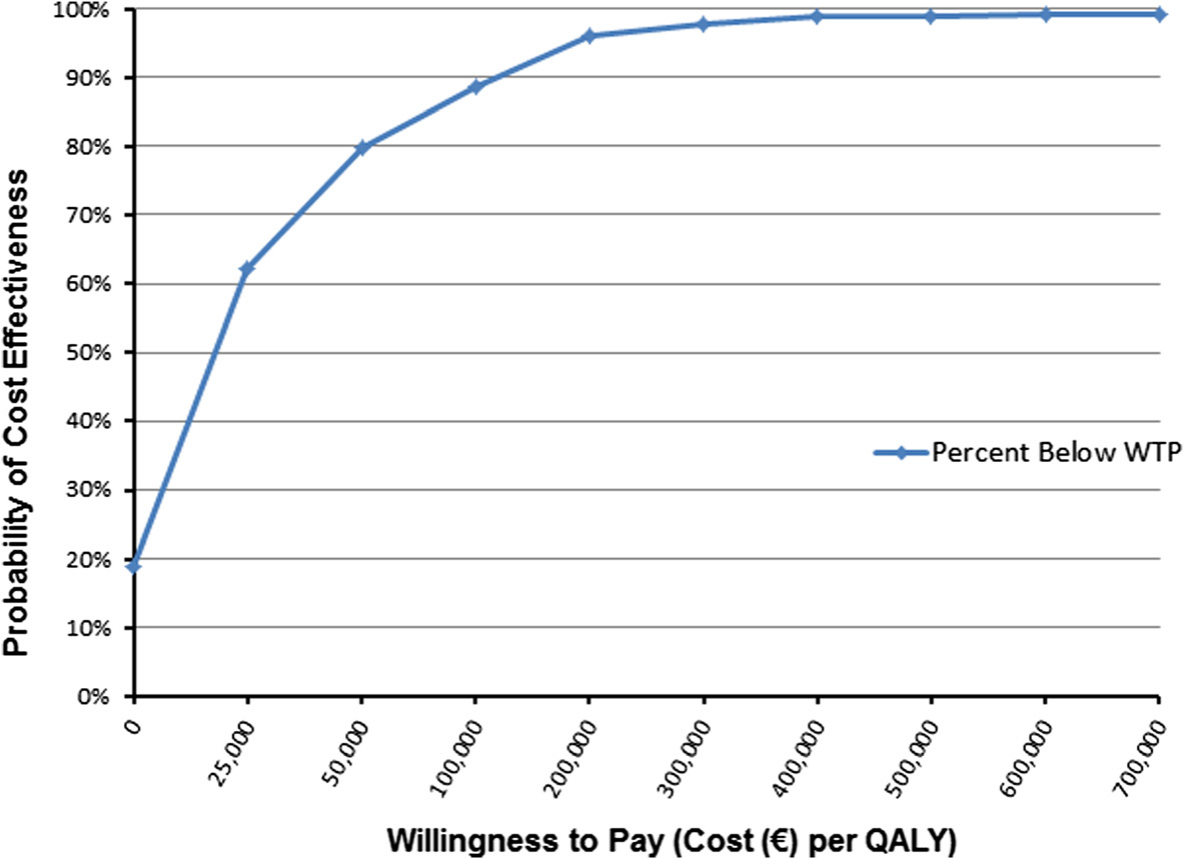
Acceptability Curve for GIOP T −2.5/2 Fracture Cohort: Teriparatide vs. Bisphosphonate Treatment.

Figure 7 shows the acceptability curve for teriparatide use versus bisphosphonate use with the GIOP T −2.5/2 fracture cohort. The acceptability curve was based on the total incremental costs and QALYs from 1,000 simulations of 1,000 patients. The acceptability curve was determined by the cumulative percentage of simulations that estimated a Cost per QALY less than a given value for willingness to pay (WTP) on the X-axis.

## Discussion

Our analysis looked at the first-line use of teriparatide against bisphosphonates and no treatment for two significant high risk osteoporosis cohorts. For both the PMO and GIOP cohorts simulated, the base case results indicate that the use of teriparatide as a first-line treatment is cost-effective at a €50,000 per QALY ICER threshold. These results included both teriparatide treatment versus no treatment and teriparatide treatment compared to bisphosphonate treatment.

One-way sensitivity analysis of the model suggests that cost per QALY estimates for teriparatide versus bisphosphonate treatment will remain under the €50,000 threshold through a wide variation of key parameters. The only case where the €50,000 threshold was exceeded was where the loss of teriparatide efficacy followed a linear decline after treatment similar to bisphosphonate (equal to the duration of therapy). That rate of post treatment efficacy decline was substantially quicker than indicated by Lindsay. Its use in this analysis is to highlight the importance of post treatment efficacy offset and also to suggest an area where further research may be warranted to extend Lindsay’s results to longer post treatment time horizons.

Probabilistic sensitivity analyses results produced by the model indicated that teriparatide was cost effective at a €50,000 per QALY threshold in over 74% of the replications simulated for both cohorts. From these results, two particular observations stand out:

1. The percentage of simulations where the estimated patients’ QALYs decreased with teriparatide usage was 0.6% for the PMO T-3.0/2 fracture cohort and 0.2% for the GIOP T-2.5/2 fracture cohort. In these cases, the mean QALY loss was less than - 0.03 QALYs for both cohorts.

2. For the PMO T-3.0/2 fracture cohort, the model estimated a cost savings and improved patient QALYs with teriparatide in 16.5% of the simulations. For the GIOP T-2.5/2 fracture cohort, the model estimated a cost savings and improved patient QALYs in 18.8% of the simulations.

The PSA results from both cohorts suggest that (using QALYs as a proxy) it is unlikely for teriparatide to have worse outcomes versus bisphosphonate for groups of 1000 patients, and if so the inferiority will be of small magnitude. Consider the results in Figures 3 and 6 in comparison to another treatment with identical mean values for cost and QALYs, but with a wider range of positive and (larger) negative QALYs. Without explicitly understanding the causal relationships responsible for those worst case (low QALY) scenarios, a decision maker may be wise (as a matter of policy) to prefer options that reduce estimated patient QALY risk.

The second observation relates to the PSA simulations where both costs are reduced and QALYs improved with teriparatide treatment versus bisphosphonate. These results beg a further analysis: is there some cohort characteristic or combination of characteristics (e.g., T-score, gender, etc.) that has a statistically greater chance to generate these most favourable outcomes? Identification of those characteristics (if they exist) might identify a patient sub group (or groups), which could lead to a more selective use of both teriparatide and bisphosphonate with lower costs per QALY for the entire cohort.

The strength of this model is the level of detail it encompasses, particularly with respect to the generation of fractures within the cohorts selected for analysis. The model provides for significant flexibility to represent initial fractures, second fractures, and fracture related mortality by patient age, BMD T-score, and gender. An additional strength of the model is its ability to be configured quickly for probabilistic and one way sensitivity analysis. However, the level of data detail required for this model also provides a drawback: data preparation, meta-analysis, and verification for a new series of analysis are non-trivial.

A subtle point related to this analysis is that it uses a given initial BMD T-score for all patients beginning treatment. For both PMO cohorts, the T-score was −3.0. In reality, a cohort of 69 year old PMO patients will have a range of BMD T-scores worse than this minimum entry point. The impact is that the cost effectiveness of the most efficacious treatment (teriparatide) will be underestimated as the number of fractures prevented was estimated on a minimum condition.

Previous cost effectiveness studies of teriparatide treatment for PMO patients in Sweden have been published by Lundkvist [14] and Borgström [29]. In the Lundkvist study, a Monte Carlo simulation was used to model a cohort of 69 year old PMO patients with a BMD T score of −3.0. Two cohorts were evaluated, one with an historic vertebral fracture and one with a recent vertebral fracture and an historic vertebral fracture. The analysis compared the cost effectiveness of an 18 month regimen of teriparatide treatment against calcium and vitamin D use (defined as no treatment).

The results presented by Lundkvist differ from those presented here. Both Lundkvist and our results indicate teriparatide treatment as a cost effective option versus no treatment for similar PMO cohorts (at a €50,000 per QALY threshold). Our estimates of ICERs, however, are significantly more favourable to teriparatide. An initial comparison of the models suggests that drug costs and post treatment efficacy (the phase out period of fracture protection) are primarily responsible for these differences. Rerunning our model using the Lundkvist data for these inputs on the PMO cohort with a T-score of −3.0 and historical vertebral fracture, we estimated similar incremental costs (€4,547 vs. €5,219), QALYs (0.07 vs. 0.08), and ICERs (€64,644 vs. €64,144) to those reported by Lundkvist. The results from the PMO cohort with T-score of −3.0 and historical and recent vertebral fracture were again similar between our model and the Lundkvist model (e.g., ICERs were €19,091 and €20,301 in the two models, respectively).

Borgström reports the cost effectiveness of teriparatide against no treatment for a similar (but not identical) Swedish PMO cohort to that evaluated here. Their analysis used a Markov model to estimate costs and outcomes in 6 month cycles. Our review indicated differences in treatment discontinuation and fracture related utilities used by Borgström that would result in the lower ICER for teriparatide versus no treatment reported in their base case analysis. Regardless of these specific differences, however, both models indicate that teriparatide is cost effective at a €50,000 per QALY threshold versus no treatment for the similar PMO cohorts evaluated.

Subsequent to the work reported in this paper, Peasgood [30] provided an extensive review of osteoporosis related health state utilities. The fracture utility values used in our model were all higher than the reference case multipliers presented in Peasgood (Table 4) for similar fractures. The impact is that QALY estimates (and hence the ICERs) generated by our model were less favorable to treatments that reduce fractures than a model using the Peasgood reference case multipliers.

A limitation of this analysis is that teriparatide is compared only to a basket of bisphosphonate treatments and to no treatment; though other osteoporosis treatment interventions are commonly prescribed. Additionally, in order to analyse patient populations who potentially would receive treatment with teriparatide, the PMO populations modeled were more severe than many patients diagnosed with osteoporosis. The analyses only reflect patient populations with the specific characteristics modeled, and thus do not reflect the actual demographic and epidemiologic distribution in the PMO and GIOP populations.

A geographical observation is also important in interpreting these results: Sweden has a high incidence of osteoporosis related fractures. While a direct translation of these results with other Nordic countries may be reasonably considered, some care should be applied in generalizing these results elsewhere.

## Conclusions

The results from this study demonstrate that there are high-risk osteoporosis patient cohorts where teriparatide use as a first-line agent is a cost-effective treatment option compared to bisphosphonates or to no treatment. These findings may provide useful information for clinical decision making in the management of osteoporosis.

## Abbreviations

BMD: Bone mineral density; CT: Computed tomography; DXA: Dual energy X-ray absorptiometry; Fx: fracture; GIOP: Glucocorticoid-induced osteoporosis; GP: General practitioner; ICER: Incremental cost-effectiveness ratios; PMO: Postmenopausal osteoporosis; PSA: Probabilistic sensitivity analysis; QALY: Quality adjusted life year; Rx: Treatment; SD: Standard deviation; SEK: Swedish Krona.

## Competing interests

The model development and analysis work reported in this paper was funded by Eli Lilly & Company (Lilly), Indianapolis, Indiana, USA. Lilly has reviewed this manuscript to ensure that no confidential information was disclosed. The model, results, analyses, and conclusions presented in this manuscript were developed solely by the authors. Medical Decision Modeling Inc. (MDM) is a contract research organization that performs pharmacoeconomic research to evaluate current and prospective treatments for medical conditions. Lee J Smolen and Robert W Klein are equity partners of MDM.

## Authors’ contributions

LJ S was the project leader for this study and was a primary contributor in the design of the model, data specification, results analysis, and manuscript review. TMK was a primary contributor to model design, model data acquisition and meta-analysis, programming, and methodology review. RWK was responsible for analysis support, manuscript development, and final manuscript review. DRM was the primary author of the manuscript, as well as performing some post project analysis specifically for the paper. All authors read and approved the final manuscript.

## Pre-publication history

The pre-publication history for this paper can be accessed here:

http://www.biomedcentral.com/1471-2474/13/213/prepub

## Supplementary Material

Additional file 1 **Appendix A, Table A1 - Model Input Data.** Table A2 – PSA Distribution Parameters. Click here for file

Additional file 2 **Appendix B - Detailed Simulation Results.** Table B1 – Results for PMO Patients (100% Female), age 69 years, T-Score = -3.0 with an historical vertebral + incident vertebral fracture. Table B2 – Results for PMO Patients (100% Female), age 69 years, T-Score = -3.0 with an historical vertebral fracture. Table B3 – Results for GIOP Patients (80% Female), age 69 years, T-Score = -2.5 with an historical vertebral + incident vertebral fracture. Table B4 – Results for GIOP Patients (80% Female), age 69 years, T-Score = -2.5 with an historical vertebral fracture. Click here for file
