# Supplementary material for: The cost effectiveness of teriparatide as a first-line treatment for glucocorticoid-induced and postmenopausal osteoporosis patients in Sweden
Source: BMC Musculoskelet Disord. 2012 Oct 30;13:213. doi: 10.1186/1471-2474-13-213 (PMC3545974; doi:10.1186/1471-2474-13-213)
Supplement: Additional file 1 — Appendix A, Table A1 - Model Input Data. Table A2 – PSA Distribution Parameters. [file 1471-2474-13-213-S1.doc]

**Appendix A**

**Table A1 - Model Input Data**

**Abbreviations:** Rx – Prescription FX-Fracture

| **Parameter** | **Value** | | | **Reference(s)** |
| --- | --- | --- | --- | --- |
| Teriparatide – Average Daily Cost | 14.74 € | | | [15] |
| Alendronate –Average Daily Cost | 0.42 € | | |
| Length of Treatment Cycle - Months | 18 | | |  |
| Rx Side Effects & Ancillary Costs | | | | [17] |
| Months 1 - 6 | 201 € | | |  |
| Months 7 -12 | 194 € | | |  |
| Months 13 - 18 | 201 € | | |  |
| Annual Fx Rates Per Person |  | | | [2, 13] |
| **Male** | **Hip** | **Vertebral** | **Wrist** |  |
| Age 52 | 0.00053 | 0.00100 | 0.00156 |  |
| Age 57 | 0.00041 | 0.00100 | 0.00070 |  |
| Age 62 | 0.00150 | 0.00147 | 0.00076 |  |
| Age 67 | 0.00257 | 0.00247 | 0.00089 |  |
| Age 72 | 0.00457 | 0.00463 | 0.00220 |  |
| Age 77 | 0.00855 | 0.00555 | 0.00144 |  |
| Age 82 | 0.01369 | 0.00545 | 0.00314 |  |
| Age 87 | 0.02019 | 0.00856 | 0.00195 |  |
| **Female** | **Hip** | **Vertebral** | **Wrist** |  |
| Age 52 | 0.00053 | 0.00117 | 0.00390 |  |
| Age 57 | 0.00055 | 0.00127 | 0.00408 |  |
| Age 62 | 0.00180 | 0.00212 | 0.00522 |  |
| Age 67 | 0.00286 | 0.00329 | 0.00592 |  |
| Age 72 | 0.00486 | 0.00583 | 0.00747 |  |
| Age 77 | 0.01151 | 0.00761 | 0.00785 |  |
| Age 82 | 0.01799 | 0.00770 | 0.00981 |  |
| Age 87 | 0.02973 | 0.01263 | 0.01025 |  |
| Fx Given Fx Rates (RR of Fx Given Fx by Age) |  |  |  | [13, 14] |
|  | **<75** | **75-84** | **>85** |  |
| Hip Fx Given Hip Fx | 2.3 | 2.3 | 2.3 |  |
| Vert. Fx (Clinical) Given Hip Fx | 2.5 | 2.5 | 2.5 |  |
| Wrist Fx Given Hip Fx | 1.4 | 1.4 | 1.4 |  |
|  |  |  |  |  |
|  | **<75** | **75-84** | **>85** |  |
| Hip Fx Given Vert. Fx | 2.3 | 2.3 | 2.3 |  |
| Vert. Fx (Clinical) Given Vert. Fx | 4.4 | 4.4 | 4.4 |  |
| Wrist Fx Given Vert. Fx | 1.4 | 1.4 | 1.4 |  |
|  |  |  |  |  |
|  | **<75** | **75-84** | **>85** |  |
| Hip Fx Given Wrist Fx | 1.9 | 1.9 | 1.9 |  |
| Vert. Fx (Clinical) Given Wrist Fx | 1.7 | 1.7 | 1.7 |  |
| Wrist Fx Given Wrist Fx | 3 | 3 | 3 |  |
|  |  | | |  |
| Years Post Fx Impact | 5 | | |  |
| Maximum Number of Hip Fxs | 4 | | |  |
| Multiple Fx Increased Fx Risk % | 100% | | |  |

**Table A1 (continued) - Model Input Data**

| **Parameter** | | **Value** | | | | **Reference(s)** | |
| --- | --- | --- | --- | --- | --- | --- | --- |
| Proportion of Mortality due to Fx | |  | | | | [13] | |
|  | | **Male** | | **Female** | |  | |
| through Age 50 | | 0 | | 0 | |  | |
| through Age 55 | | 0 | | 0 | |  | |
| through Age 60 | | 0 | | 0 | |  | |
| through Age 65 | | 0.007 | | 0.0128 | |  | |
| through Age 70 | | 0.0081 | | 0.0082 | |  | |
| through Age 75 | | 0.0123 | | 0.0171 | |  | |
| through Age 80 | | 0.0163 | | 0.0188 | |  | |
| through Age 85 | | 0.023 | | 0.0268 | |  | |
| through Age 90 and beyond | | 0.0334 | | 0.0375 | |  | |
| Relative Risk Mortality Post Fx | |  | |  | | [19, 20] | |
|  | | **Post Hip Fx** | | | **Post Vertebral Fx** |  | |
| Year 1 | | 2.5 | | | 2.5 |  | |
| Year 2 | | 2.1 | | | 1.3 |  | |
| Year 3 | | 1.8 | | | 1.3 |  | |
| Year 4 | | 1.6 | | | 1.3 |  | |
| Year 5 | | 1.4 | | | 1.3 |  | |
| Year 6 | | 1.2 | | | 1 |  | |
| Year 7 | | 1 | | | 1 |  | |
| Relative Risk Fracture Associated with Glucocorticoid Use | |  | | |  | [13] | |
|  | | **Wrist Fx** | | | **Hip Fracture & Clinical Vertebral Fx** |  | |
| Age 50 | | 2.63 | | | 4.42 |  | |
| 55 | | 2.32 | | | 4.15 |  | |
| 60 | | 2.00 | | | 3.71 |  | |
| 65 | | 1.81 | | | 2.98 |  | |
| 70 | | 1.76 | | | 2.44 |  | |
| 75 | | 1.70 | | | 2.22 |  | |
| 80 | | 1.59 | | | 2.13 |  | |
| 85 | | 1.71 | | | 2.48 |  | |
| Hip Fx Direct Costs (€) |  | |  | | | | [14, 30] |
|  | **Initial** | | **Subsequent** | | | |  |
| Ages < 60 | 7,267 € | | 3,056 € | | | |  |
| Ages 60-69 | 8,122 € | | 3,056 € | | | |  |
| Ages 70-79 | 16,641 € | | 3,056 € | | | |  |
| Ages 80+ | 24,475 € | | 3,056 € | | | |  |
| Vertebral Fx (Clinical) Direct Costs (€) |  | |  | | | | [14, 30] |
|  | **Initial** | | **Subsequent** | | | |  |
| Ages < 60 | 3,409 € | | 304 € | | | |  |
| Ages 60-69 | 3,409 € | | 304 € | | | |  |
| Ages 70-79 | 3,409 € | | 304 € | | | |  |
| Ages 80+ | 3,409 € | | 304 € | | | |  |

**Table A1 (continued) - Model Input Data**

| **Parameter** | **Value** | | | | **Reference(s)** |
| --- | --- | --- | --- | --- | --- |
| Wrist Fx (Clinical) Direct Costs (€) |  | |  | | [14, 30] |
|  | **Initial** | | **Subsequent** | |  |
| Ages < 60 | 2,359 € | | 0 € | |  |
| Ages 60-69 | 2,359 € | | 0 € | |  |
| Ages 70-79 | 2,359 € | | 0 € | |  |
| Ages 80+ | 2,359 € | | 0 € | |  |
| Initial Utility by Age |  | |  | | [21] |
|  | **Male** | | **Female** | |  |
| through Age 50 | 0.84 | | 0.82 | |  |
| through Age 55 | 0.84 | | 0.82 | |  |
| through Age 60 | 0.81 | | 0.79 | |  |
| through Age 65 | 0.81 | | 0.79 | |  |
| through Age 70 | 0.76 | | 0.71 | |  |
| through Age 75 | 0.76 | | 0.71 | |  |
| through Age 80 | 0.65 | | 0.62 | |  |
| through Age 85 | 0.65 | | 0.62 | |  |
| Utility Multipliers |  | |  | | [13], Table 30 |
|  | **1st Year** | | **Subsequent Years** | |  |
| Hip Fx | 0.792 | | 0.813 | |  |
| Vertebral Fx (Clinical) | 0.626 | | 0.909 | |  |
| Wrist Fx | 0.977 | | 0.999 | |  |
|  |  | | | |  |
| % of Prev Fx Utility Used | 25% | | | |  |
| Discount Rate | 3% | | | |  |
| Discount Utility Rate | 3% | | | |  |
| Teriparatide – Fx Rel. Risks (Hip Fx, Vert Fx, Other Fx) |  |  | |  | [4–7] |
|  | **Hip** | **Vertebral** | | **Non-Vert** |  |
| Treatment: Months 1-6 | 0.47 | 0.17 | | 0.47 |  |
| Treatment: Months 7-12 | 0.47 | 0.17 | | 0.47 |  |
| Treatment: Months 13-18 | 0.47 | 0.17 | | 0.47 |  |
| Post Treatment: Months 19-24 | 0.73 | 0.43 | | 0.73 |  |
| Post Treatment: Months 25-30 | 0.73 | 0.43 | | 0.73 |  |
| Post Treatment: Months 31-36 | 0.73 | 0.525 | | 0.73 |  |
| Post Treatment: Months 37-42 | 0.73 | 0.715 | | 0.73 |  |
| Post Treatment: Months 42-48 | 0.775 | 0.905 | | 0.775 |  |
| Post Treatment: Months 49-54 | 0.865 | 1 | | 0.865 |  |
| Post Treatment: Months 55-60 | 0.955 | 1 | | 0.955 |  |
| Bisphosphonate – Fx Rel. Risks (Hip Fx, Vert Fx, Other Fx) |  |  | |  | [13] |
|  | **Hip** | **Vertebral** | | **Non-Vert** |  |
| Treatment: Months 1-6 | 0.61 | 0.57 | | 0.81 |  |
| Treatment: Months 7-12 | 0.61 | 0.57 | | 0.81 |  |
| Treatment: Months 13-18 | 0.61 | 0.57 | | 0.81 |  |
| Post Treatment: Months 19-36 | Linear phase out over treatment duration | | | | [30] |
| SEK to € Conversion Rate | 9.0335 | | | | [16] |

**Table A1 (continued) - Model Input Data**

| **Parameter** | **Value** | | **Reference(s)** |
| --- | --- | --- | --- |
| Treatment Continuation |  |  |  |
|  |  |  |  |
| Teriparatide | Months 1-6 | 0.93 | [22] |
|  | Months 7-12 | 0.83 |  |
|  | Months 13-18 | 0.70 |  |
| Bisphosphonate | Months 1-6 | 0.76 | [23] |
|  | Months 7-12 | 0.58 |  |
|  | Months 13-18 | 0.45 |  |

**Table A2 –** PSA Distribution Parameters

| **Parameter** |  | **Value** |
| --- | --- | --- |
| Hip Fracture Costs (Initial) |  |  |
| Distr. Type |  | Lognormal |
| Mean | Ages < 60 | 7,267 € |
|  | Ages 60-69 | 8,122 € |
|  | Ages 70-79 | 16,6641 € |
|  | Ages 80+ | 24,475 € |
| St. Dev. | Ages < 60 | 5,267 € |
|  | Ages 60-69 | 6,401 € |
|  | Ages 70-79 | 13,115 € |
|  | Ages 80+ | 18,289 € |
| Minimum | Ages < 60 | 3,634 € |
|  | Ages 60-69 | 4,061 € |
|  | Ages 70-79 | 8,321 € |
|  | Ages 80+ | 12,238 € |
| Maximum | Ages < 60 | 14,534 € |
|  | Ages 60-69 | 16,244 € |
|  | Ages 70-79 | 33,282 € |
|  | Ages 80+ | 48,950 € |
| Hip Fracture Costs (Subsequent) |  |  |
| Distr. Type |  | Lognormal |
| Mean |  | 3,056 € |
| St. Dev. |  | 2,408 € |
| Minimum |  | 1,528 € |
| Maximum |  | 6,112 € |
| Vertebral Fracture Costs (Initial) |  |  |
| Distr. Type |  | Lognormal |
| Mean |  | 3,409 € |
| St. Dev. |  | 2,687 € |
| Minimum |  | 1,705 € |
| Maximum |  | 6,818 € |
| Vertebral Fracture Costs (Subsequent) |  |  |
| Distr. Type |  | Lognormal |
| Mean |  | 304 € |
| St. Dev. |  | 240 € |
| Minimum |  | 152 € |
| Maximum |  | 608 € |
| Wrist Fracture Costs |  |  |
| Distr. Type |  | Lognormal |
| Mean |  | 2,359 € |
| St. Dev. |  | 1,859 € |
| Minimum |  | 1,180 € |
| Maximum |  | 4,718 € |
| Teriparatide Hip Fracture Relative Risk |  |  |
| Distr. Type |  | Beta |
| Mean |  | 0.47 |
| St. Dev. |  | 0.108 |
| Minimum |  | 0.25 |
| Maximum |  | 0.90 |
| Teriparatide Vertebral Fracture Relative Risk |  |  |
| Distr. Type |  | Beta |
| Mean |  | 0.17 |
| St. Dev. |  | 0.063 |
| Minimum |  | 0.07 |
| Maximum |  | 0.45 |
| Teriparatide Wrist Fracture Relative Risk |  |  |
| Distr. Type |  | Beta |
| Mean |  | 0.47 |
| St. Dev. |  | 0.108 |
| Minimum |  | 0.25 |
| Maximum |  | 0.90 |
| Hip Fracture Relative Disutility (Year of Fracture) |  |  |
| Distr. Type |  | Beta |
| Mean |  | 0.792 |
| St. Dev. |  | 0.034 |
| Minimum |  | 0.696 |
| Maximum |  | 0.899 |
| Hip Fracture Relative Disutility (Subsequent Years) |  |  |
| Distr. Type |  | Beta |
| Mean |  | 0.813 |
| St. Dev. |  | 0.032 |
| Minimum |  | 0.714 |
| Maximum |  | 0.905 |
| Vertebral Fracture Relative Disutility (Year of Fracture) |  |  |
| Distr. Type |  | Beta |
| Mean |  | 0.626 |
| St. Dev. |  | 0.052 |
| Minimum |  | 0.536 |
| Maximum |  | 0.845 |
| Vertebral Fracture Relative Disutility (Subsequent Years) |  |  |
| Distr. Type |  | Beta |
| Mean |  | 0.909 |
| St. Dev. |  | 0.016 |
| Minimum |  | 0.857 |
| Maximum |  | 0.952 |
| Wrist Fracture Relative Disutility (Year of Fracture) |  |  |
| Distr. Type |  | Beta |
| Mean |  | 0.977 |
| St. Dev. |  | 0.004 |
| Minimum |  | 0.964 |
| Maximum |  | 0.988 |
| Wrist Fracture Relative Disutility (Subsequent Years) |  |  |
| Distr. Type |  | Beta |
| Mean |  | 0.999 |
| St. Dev. |  | 0.002 |
| Minimum |  | 0.988 |
| Maximum |  | 1.000 |
