# Supplementary material for: The cost effectiveness of teriparatide as a first-line treatment for glucocorticoid-induced and postmenopausal osteoporosis patients in Sweden
Source: BMC Musculoskelet Disord. 2012 Oct 30;13:213. doi: 10.1186/1471-2474-13-213 (PMC3545974; doi:10.1186/1471-2474-13-213)
Supplement: Additional file 2 — Appendix B - Detailed Simulation Results. Table B1 – Results for PMO Patients (100% Female), age 69 years, T-Score = -3.0 with an historical vertebral + incident vertebral fracture. Table B2 – Results for PMO Patients (100% Female), age 69 years, T-Score = -3.0 with an historical vertebral fracture. Table B3 – Results for GIOP Patients (80% Female), age 69 years, T-Score = -2.5 with an historical vertebral + incident vertebral fracture. Table B4 – Results for GIOP Patients (80% Female), age 69 years, T-Score = -2.5 with an historical vertebral fracture. [file 1471-2474-13-213-S2.doc]

**Appendix B - Detailed Simulation Results**

**Table B1 – Results for PMO Patients (100% Female), age 69 years, T-Score = -3.0 with an historical vertebral + incident vertebral fracture**

| **Fractures per 1,000 Patients (95% CI)** | | | | |
| --- | --- | --- | --- | --- |
|  | **Hip Fractures** | **Vertebral Fractures** | | **Wrist Fractures** |
| No Treatment | 1,743  (1,735 – 1,749) | 1,188  (1,180 – 1,196) | | 337  (333 – 340) |
| Bisphosphonates | 1,714  (1,706 – 1,721) | 1,133  (1,125 – 1,141) | | 331  (328 – 334) |
| Teriparatide | 1,648  (1,641 – 1,655) | 967  (960 – 974) | | 304  (301 – 307) |
| **Incremental Fractures avoided per 1,000 Patients (95% CI)** | | | | |
|  | **Hip Fractures** | **Vertebral Fractures** | | **Wrist Fractures** |
| Teriparatide vs. No Treatment | 95  (92 – 98) | 221  (218 – 224) | | 32  (30 – 34) |
| Teriparatide vs. Bisphosphonates | 66  (64 – 69) | 166  (163 – 169) | | 27  (25 – 28) |
| **Cost, QALYs and Life Years per 1,000 Patients (95% CI)** | | | | |
|  | **Costs** | **QALYs** | | **Life Years** |
| No Treatment | €57,802,281  (€57,513,657 – €58,008,250) | 6,305  (6,285 – 6,323) | | 14,317  (14,273 – 14,362) |
| Bisphosphonates | €56,339,268  (€56,067,149 – €56,560,080) | 6,361  (6,342 – 6,380) | | 14,347  (14,305 –14,393) |
| Teriparatide | €58,916,532  (€58,690,565 – €59,110,355) | 6,494  (6,475 – 6,512) | | 14,449  (14,404 – 14,493) |
| **Incremental Cost Effectiveness per 1,000 Patients (95% CI)** | | | | |
|  | **Costs** | **QALYs** | | **Life Years** |
| Teriparatide vs. No Treatment | €1,114,251  (€964,206 – €1,242,530) | 189  (185-193) | | 132  (123 – 141) |
| Teriparatide vs. Bisphosphonates | €2,577,265  (€2,470,001 – €2,689,403) | 133  (129-136) | | 102  (95 – 109) |
| **Cost Effectiveness Ratios (95% CI)** | | | | |
| Teriparatide vs. No Treatment | €5,897 / QALY | | (€5,128 - €6,612) | |
| Teriparatide vs. Bisphosphonates | €19,371 / QALY | | (€18,413 - €20,424) | |

**Appendix B - Detailed Simulation Results (continued)**

**Table B2 – Results for PMO Patients (100% Female), age 69 years, T-Score = -3.0 with an historical vertebral fracture**

| **Fractures per 1,000 Patients (95% CI)** | | | | |
| --- | --- | --- | --- | --- |
|  | **Hip Fractures** | **Vertebral Fractures** | | **Wrist Fractures** |
| No Treatment | 1,614  (1,607 – 1,620) | 942  (935 – 949) | | 311  (308 – 314) |
| Bisphosphonates | 1,591  (1,584 – 1,598) | 905  (899 – 912) | | 306  (303 – 309) |
| Teriparatide | 1,541  (1,534 – 1,547) | 797  (790 – 803) | | 284  (280 – 287) |
| **Incremental Fractures avoided per 1,000 Patients (95% CI)** | | | | |
|  | **Hip Fractures** | **Vertebral Fractures** | | **Wrist Fractures** |
| Teriparatide vs. No Treatment | 73  (70 – 75) | 145  (142 – 149) | | 27  (26 – 29) |
| Teriparatide vs. Bisphosphonates | 51  (48 – 53) | 108  (105 – 111) | | 22  (21 – 23) |
| **Cost, QALYs and Life Years per 1,000 Patients (95% CI)** | | | | |
|  | **Costs** | **QALYs** | | **Life Years** |
| No Treatment | €50,319,914  (€50,073,975 – €50,540,818) | 6,534  (6,514 – 6,553) | | 14,501  (14,455 – 14,547) |
| Bisphosphonates | €49,322,393  (€49,088,090 – €49,524,637) | 6,576  (6,555 – 6,594) | | 14,526  (14,482 – 14,571) |
| Teriparatide | €52,922,696  (€52,713,307 – €53,096,649) | 6,673  (6,653 – 6,692) | | 14,603  (14,558 – 14,647) |
| **Incremental Cost Effectiveness per 1,000 Patients (95% CI)** | | | | |
|  | **Costs** | **QALYs** | | **Life Years** |
| Teriparatide vs. No Treatment | €2,602,782  (€2,471,035 – €2,740,764) | 139  (135 – 143) | | 102  (95 – 110) |
| Teriparatide vs. Bisphosphonates | €3,600,303  (€3,490,963 – €3,714,087) | 97  (94 – 100) | | 77  (70 – 84) |
| **Cost Effectiveness Ratios (95% CI)** | | | | |
| Teriparatide vs. No Treatment | €18,701 / QALY | | (€17,612 – €20,062) | |
| Teriparatide vs. Bisphosphonates | €36,995 / QALY | | (€35,252 – €38,944) | |

**Appendix B - Detailed Simulation Results (continued)**

**Table B3 – Results for GIOP Patients (80% Female), age 69 years, T-Score = -2.5 with an historical vertebral + incident vertebral fracture**

| **Fractures per 1,000 Patients (95% CI)** | | | | |
| --- | --- | --- | --- | --- |
|  | **Hip Fractures** | **Vertebral Fractures** | | **Wrist Fractures** |
| No Treatment | 1,951  (1,944 – 1,958) | 1,750  (1,740 – 1,761) | | 388  (384 – 393) |
| Bisphosphonates | 1,921  (1,913 – 1,929) | 1,674  (1,664 – 1,684) | | 381  (377 – 386) |
| Teriparatide | 1,858  (1,851 – 1,866) | 1,447  (1,437 – 1,457) | | 351  (347 – 356) |
| **Incremental Fractures avoided per 1,000 Patients (95% CI)** | | | | |
|  | **Hip Fractures** | **Vertebral Fractures** | | **Wrist Fractures** |
| Teriparatide vs. No Treatment | 93  (90 – 96) | 304  (300 – 309) | | 36  (35 – 38) |
| Teriparatide vs. Bisphosphonates | 63  (60 – 65) | 227  (223 – 231) | | 30  (28 – 31) |
| **Cost, QALYs and Life Years per 1,000 Patients (95% CI)** | | | | |
|  | **Costs** | **QALYs** | | **Life Years** |
| No Treatment | €64,406,731  (€64,115,539 – €64,630,365) | 5,868  (5,851 – 5,891) | | 13,335  (13,294 – 13,382) |
| Bisphosphonates | €62,772,191  (€62,525,226 – €62,989,558) | 5,934  (5,917 – 5,957) | | 13,371  (13,332 – 13,417) |
| Teriparatide | €65,134,023  (€64,892,471 – €65,342,781) | 6,090  (6,072 – 6,112) | | 13,488  (13,448 – 13,533) |
| **Incremental Cost Effectiveness per 1,000 Patients (95% CI)** | | | | |
|  | **Costs** | **QALYs** | | **Life Years** |
| Teriparatide vs. No Treatment | €727,291  (€602,032 – €846,863) | 222  (219 – 226) | | 153  (144 – 163) |
| Teriparatide vs. Bisphosphonates | €2,361,832  (€2,260,974 – €2,454,497) | 156  (153 – 159) | | 117  (109 – 125) |
| **Cost Effectiveness Ratios (95% CI)** | | | | |
| Teriparatide vs. No Treatment | €3,271 / QALY | | (€2,691 – €3,853) | |
| Teriparatide vs. Bisphosphonates | €15,155 / OALY | | (€14,406 – €15,881) | |

**Appendix B - Detailed Simulation Results (continued)**

**Table B4 – Results for GIOP Patients (80% Female), age 69 years, T-Score = -2.5 with an historical vertebral fracture**

| **Fractures per 1,000 Patients (95% CI)** | | | | |
| --- | --- | --- | --- | --- |
|  | **Hip Fractures** | **Vertebral Fractures** | | **Wrist Fractures** |
| No Treatment | 1,883  (1,875 – 1890) | 1,558  (1,548 – 1,567) | | 374  (370 – 379) |
| Bisphosphonates | 1,856  (1,847 – 1,863) | 1,494  (1,484 – 1,503) | | 368  (363 – 373) |
| Teriparatide | 1,798  (1,790 – 1,805) | 1,310  (1,301 – 1,319) | | 340  (336 – 344) |
| **Incremental Fractures avoided per 1,000 Patients (95% CI)** | | | | |
|  | **Hip Fractures** | **Vertebral Fractures** | | **Wrist Fractures** |
| Teriparatide vs. No Treatment | 85  (82 – 87) | 248  (244 – 253) | | 34  (33 – 36) |
| Teriparatide vs. Bisphosphonates | 57  (55 – 60) | 184  (180 – 188) | | 28  (26 – 29) |
| **Cost, QALYs and Life Years per 1,000 Patients (95% CI)** | | | | |
|  | **Costs** | **QALYs** | | **Life Years** |
| No Treatment | €60,112,779  (€59,810,148 – €60,309,216) | 6,015  (5,998 – 6,037) | | 13,450  (13,407 – 13,498) |
| Bisphosphonates | €58,707,306  (€58,435,913 – €58,911,645) | 6,074  (6,055 – 6,096) | | 13,485  (13,443 – 13,532) |
| Teriparatide | €61,542,647  (€61,291,100 – €61,741,988) | 6,210  (6,192 – 6,229) | | 13,590  (13,549 – 13,634) |
| **Incremental Cost Effectiveness per 1,000 Patients (95% CI)** | | | | |
|  | **Costs** | **QALYs** | | **Life Years** |
| Teriparatide vs. No Treatment | €1,429,868  (€1,318,486 – €1,549,621) | 195  (192 – 199) | | 140  (132 – 148) |
| Teriparatide vs. Bisphosphonates | €2,835,341  (€2,738,615 – €2,930,202) | 136  (133 – 139) | | 106  (99 – 113) |
| **Cost Effectiveness Ratios (95% CI)** | | | | |
| Teriparatide vs. No Treatment | €7,330 / QALY | | (€6,650 – €8,062) | |
| Teriparatide vs. Bisphosphonates | €20,826 / QALY | | (€19,831 – €21,854) | |
